# IL-6 knockdown anti-CD19 CAR-T cells (ssCART-19) for patients with relapsed or refractory acute lymphoblastic leukemia: phase 1 trial

**DOI:** 10.1038/s41408-025-01397-4

**Published:** 2025-10-27

**Authors:** Sheng-Li Xue, Mei-Jing Liu, Chong-Sheng Qian, Su-Ning Chen, Hui-Ying Qiu, Li-Qing Kang, Hai-Ping Dai, Wen-Jie Gong, Li-Yun Chen, Zhen Yao, Ming-Zhu Xu, Hai-Xia Zhou, Xiao-Fei Yang, Qian Wu, Xue-Qing Dou, Jian Zhang, Yin Liu, Qing-Ya Cui, Zheng Li, Yang Xu, Man Qiao, Tong-Tong Zhang, Jing-Wen Tan, Nan Xu, Ming-Hao Li, Zhou Yu, Xiao-Yan Lou, Wei Wang, Hong-Jia Zhu, Lu Qin, Lei Yu, Xiao-Wen Tang, De-Pei Wu

**Affiliations:** 1 National Clinical Research Center for Hematologic Diseases, Jiangsu Institute of Hematology, The First Affiliated Hospital of Soochow University, Suzhou, China; 2 Institute of Blood and Marrow Transplantation, Collaborative Innovation Center of Hematology, Soochow University, Suzhou, China; 3 Shanghai Unicar-Therapy Bio-Medicine Technology Co. Ltd, Shanghai, China

**Keywords:** Phase I trials, Cancer immunotherapy

**Dear Editor,**

CD19-targeted chimeric antigen receptor-modified T (CAR-T) cell therapy is a revolutionary immunotherapy for relapsed or refractory B-cell acute lymphoblastic leukemia (r/r B-ALL), with 60%–80% response rates [1]. However, cytokine release syndrome (CRS) and immune effector cell-associated neurotoxicity syndrome (ICANS) remain life-threatening toxicities in CAR-T therapy, constraining its wider clinical adoption [1–3]. In the ZUMA-3 study of KTE-X19 for r/r B-ALL, 93% of patients developed CRS, with 31% graded ≥3, and 78% developed ICANS, with 38% being grade 3 or worse [1].

Interleukin 6 (IL-6) is a key cytokine in CRS and is also implicated in ICANS, although its role in ICANS is less direct and likely involves other cytokines (e.g., IL-1, granulocyte-macrophage colony-stimulating factor [GM-CSF]) and blood–brain barrier (BBB) dysfunction [4]. Importantly, IL-6 derived from CAR-T cells may be a key initiator of monocytic IL-6 release, potentially driving severe CRS [5]. Although tocilizumab remains standard treatment for CRS, its efficacy against ICANS is limited owing to poor penetration of the BBB [3]. To address these limitations, we developed a novel anti-CD19 CAR-T cell incorporating an IL-6-targeting short hairpin RNA (shRNA), designated ssCART-19. Functionally, this modification substantially suppresses IL-6 release from monocytes while maintaining antitumor efficacy of ssCART-19 in vitro and in vivo [5].

Herein, we report initial findings of a single-center, single-arm, phase 1 trial evaluating the safety and efficacy of ssCART-19 in adults with r/r B-ALL. This study was conducted in accordance with the Declaration of Helsinki, approved by the Ethics Committee of the First Affiliated Hospital of Soochow University, and registered with ClinicalTrials.gov (NCT04825496). Written informed consent was obtained from all patients.

Eligible patients were aged 18–65 years with confirmed diagnosis of r/r B-ALL, ≥5% morphological bone marrow blasts, CD19 expression on flow cytometry, Eastern Cooperative Oncology Group performance status of 0–1, and adequate organ function. Full eligibility criteria are detailed in the Supplementary information.

Patients underwent lymphodepletion with fludarabine (30 mg/m^2^/day) and cyclophosphamide (300 mg/m^2^/day) from days −5 to −3. CAR-T cells were administered in fractional infusions of 10%, 30%, and 60% of the total dose. During the dose-escalation phase, ssCART-19 was infused at total doses of 1 × 10^6^ (low), 5 × 10^6^ (medium), or 1 × 10^7^ (high) cells/kg. In the dose expansion phase, patients received the phase 2 recommended dose, determined according to safety and efficacy profiles observed during escalation.

The primary endpoint was safety, evaluated based on the occurrence of dose-limiting toxicity (DLT) within the initial 28 days after ssCART-19 infusion. Adverse events were documented and graded according to the National Cancer Institute Common Terminology Criteria for Adverse Events Version 5.0. CRS and ICANS were graded per the American Society for Transplantation and Cellular Therapy [6]. Secondary endpoints comprised the objective response rate (ORR, defined as the proportion of patients achieving complete remission [CR] or CR with incomplete hematological recovery [CRi] by 3 months), duration of response (DOR), progression-free survival (PFS), overall survival (OS), and pharmacokinetic parameters (detailed in the Supplementary information).

All analyses were performed using IBM SPSS (v.22) and GraphPad Prism (v.9.0.0). Full methodological details are provided in the Supplementary information.

Between April 9, 2021, and October 31, 2023, a total 31 patients with r/r B-ALL were screened for eligibility. Ten patients failed screening, and 21 were consecutively enrolled and underwent apheresis. ssCART-19 was successfully manufactured for all enrolled patients. Four patients did not receive infusion owing to uncontrolled pulmonary infection (n = 2), intracerebral hemorrhage (n = 1), or disease progression (n = 1). Ultimately, 17 patients were infused with ssCART-19. Patients’ disposition is summarized in Fig. 1A.

**Fig. 1:**
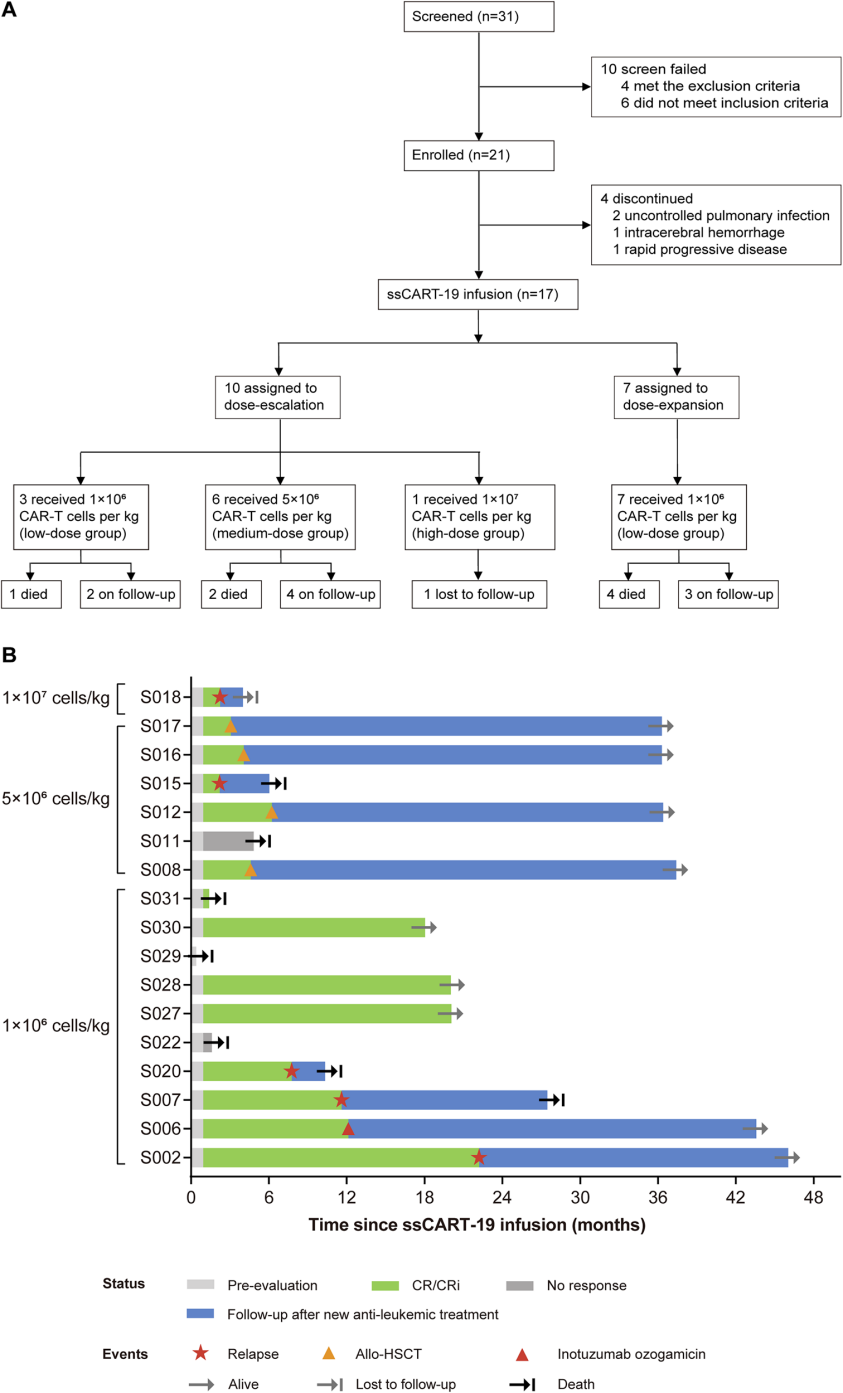
Patient disposition and clinical response to ssCART-19 infusion. **A** Flowchart of patient enrollment in the study. **B** Swimmer’s plot of individual patient clinical response and follow-up.

Baseline patient characteristics are summarized in Table 1 and Supplementary Table 1. The median participant age was 39 years (interquartile range [IQR], 20–51). Median bone marrow blast percentage was 18.0% (IQR, 5.0–53.3%), and 76.5% of patients exhibited more than 5% blasts prior to infusion. CD19 expression was ≥93.7% in leukemia blasts from 16/17 patients; one patient showed reduced expression (21.8%). Poor-risk genetics were identified in eight patients (47.1%), including mutated *TP53*, *BCR::ABL1* gene fusions, and *BCR::ABL1*-like characteristics.

**Table 1:** Baseline characteristics.

Characteristic *n* (%) / Median (range)	Patients (*N* = 17)
Median age, years	39 (20–51)
Sex	
Male	9 (52.9)
Female	8 (47.1)
Median time since diagnosis, months	17 (4–28)
ECOG performance status before ssCART-19 infusion	
0	5 (29.4)
1	12 (70.6)
Philadelphia positive	
Yes	3 (17.6)
No	14 (82.4)
Previous TKI therapy	
Yes	4 (23.5)
No	13 (76.5)
Previous stem cell transplantation	
Auto-HSCT	0 (0.0)
Allo-HSCT	2 (11.8)
No	15 (88.2)
Disease status	
Refractory	3 (17.6)
Relapsed	14 (82.4)
Number of relapses	
0	3 (17.6)
1	10 (58.9)
2	2 (11.8)
>2	2 (11.8)
Previous bridging therapy	
Yes	14 (82.4)
No	3 (17.6)
High-risk cytogenetic features	
Yes	8 (47.1)
No	9 (52.9)
Bone marrow leukemia blasts at screening	
≤5%	0 (0.0)
>5%, ≤25%	8 (47.1)
>25%, ≤50%	1 (5.9)
>50%, ≤75%	4 (23.5)
>75%	4 (23.5)
Extramedullary disease at screening	
Yes	1 (5.9)
No	16 (94.1)
CNSL disease at screening	
Yes	1 (5.9)
No	16 (94.1)

No DLT was observed among participants. All patients experienced at least one adverse event related to ssCART-19 (Supplementary Table 2). The most common adverse events grade 3 or higher were hematological: lymphocytopenia (16/17, 94.1%), neutropenia (15/17, 88.2%), leukopenia (15/17, 88.2%), anemia (12/17, 70.6%), and thrombocytopenia (10/17, 58.8%). Two patients died of septic shock caused by prolonged severe neutropenia following ssCART-19 infusion. B-cell aplasia occurred in all patients, with a median onset time of 0.0 days (IQR, 0.0–2.0) and median duration of 90.0 days (IQR, 74.5–183.8). Four patients exhibited persistent B-cell aplasia at the final follow-up visit.

CRS occurred in 13/17 patients (76.5%), with grade 3 CRS in three patients (17.6%) and no grade 4 events observed. During dose escalation, rates of grade 3 CRS were 0% (0/3), 33.3% (2/6), and 0% (0/1) in the low-, medium-, and high-dose cohorts, respectively. Following dose expansion, the grade 3 CRS rate was 10% (1/10) in the low-dose cohort, detailed in Supplementary Table 3.

All cases of CRS resolved rapidly following conventional interventions, including tocilizumab (nine patients), glucocorticoids (eight patients), or a combination of both (eight patients). Most of these patients had a high tumor burden, with median bone marrow blast percentage 49.0% (IQR, 22.0–66.5%). No neurological toxicity was observed in any patients, including the individual with central nervous system leukemia (CNSL) at screening. No correlation was found between peak cytokine levels and either CRS severity or the administered dose of ssCART-19 (Supplementary Figs. 1 and 2).

During dose escalation, all three patients (100%) in the low-dose group, four of six (66.7%) in the medium-dose group, and none (one patient, 0%) in the high-dose group achieved CR or CRi by 3 months post infusion. Based on the safety and efficacy profiles observed during dose escalation, an additional 7 patients were enrolled in the low-dose group (1×10⁶ cells/kg) as part of the expansion cohort. Following dose expansion, 11/17 patients (64.7%) attained objective remission at 3 months, comprising eight with CR (47.1%) and three with CRi (17.6%), all of whom were minimal residual disease (MRD)-negative. Individual responses and survival outcomes are illustrated in a swimmer’s plot (Fig. 1B).

With median follow-up of 20.1 months (IQR, 4.0–36.4), the median DOR was 25.8 months (95% confidence interval [CI] 4.8–46.9) for the 14 patients who achieved CR or CRi. The median DOR was 21.3 months (95% CI 5.3–37.3) for the eight patients treated with 1 × 10^6^ CAR-T cells/kg who achieved CR or CRi by day 28. The median PFS was 22.2 months (95% CI 0.0–47.0) across all dose levels and 11.6 months (95% CI 0.0–24.4) in the low-dose group. OS was not reached across all dose levels (Supplementary Fig. 3). Four patients in the medium-dose group underwent allogeneic hematopoietic stem cell transplantation (allo-HSCT) at a median 5.0 months (IQR, 3.6–7.0) post infusion.

Two patients who did not respond to ssCART-19 died due to disease progression at 1.6 months and 4.8 months post infusion. Three additional patients experienced relapse and died due to progressive disease, including one with CD19-negative relapse at 7.8 months. The patient in the high-dose group relapsed at 3.2 months and was subsequently lost to follow-up.

Expansion of CAR-T cells was detected in all patients (Supplementary Figs. 4 and 5). ssCART-19 expansion was higher in patients who achieved CR or CRi at 3 months post infusion than in non-responders (Supplementary Fig. 5C; P = 0.0279). A trend toward increased AUC_0-28d_ (area under the curve during the first 28 days following CAR-T cell infusion) was observed in patients who achieved CR or CRi (Supplementary Fig. 5D). Patients who experienced relapse had undetectable CAR-T cells at the time of disease progression.

In this phase 1 trial, ssCART-19 demonstrated an ORR of 64.7% at 3 months post infusion. CRS occurred in 76.5% of patients, with grade 3 CRS observed in 17.6% across all dose levels and 10% in the low-dose cohort, significantly lower than reported rates for conventional anti-CD19 CAR-T cell therapies [1, 7, 8]. Future direct head-to-head comparisons are warranted to validate these findings, particularly given the differences in tumor burden across study cohorts.

Numerous strategies to mitigate CRS and ICANS are reported, including tocilizumab and corticosteroids, although each has limitations including poor BBB penetration or immunosuppression [9, 10]. Whereas both GM-CSF and IL-1 are implicated in CRS pathogenesis, the clinical benefit of CAR-T cells deficient in these cytokines remains unproven [4]. In this study, patients exhibited comparable peak IL-6 levels across all dose levels, an effect attributed to IL-6 knockdown via shRNA. IL-6 peaks were still observed in some patients despite relatively favorable CRS and ICANS rates, an effect potentially related to tumor burden. Although high tumor burden is associated with severe CRS in patients receiving conventional CAR-T cell therapy [11*–*13], we did not observe an increased CRS incidence in such patients, suggesting a potential safety advantage of ssCART-19 in this population. The favorable ICANS profile and one pilot data indicate that ssCART-19 may offer a promising treatment strategy for patients with r/r B-ALL and central nervous system involvement [14].

We observed an ORR of 82.4% at day 28 and 64.7% at 3 months post infusion, accompanied by an increasing rate of MRD-negative remission. The low-dose cohort achieved an ORR of 80% at 3 months, with a favorable safety profile. Reduced CAR-T doses streamlined manufacturing by shortening production time, lowering costs, and yielding a less-differentiated CAR-T cell product [15]. Deaths resulting from severe infections and disease progression in our study underscore the critical importance of managing treatment-related toxicities and implementing bridging to allo-HSCT.

In conclusion, ssCART-19 demonstrated favorable tolerability and promising efficacy in patients with r/r B-ALL. These findings position ssCART-19 as a potential therapeutic option for patients with a high tumor burden. A multicenter phase 2 trial is currently underway to evaluate efficacy and safety of the recommended dose (1×10^6^ CAR-T cells/kg) in an expanded cohort, with extended survival follow-up ongoing.

## Supplementary information

Supplementary Information

## Data Availability

The datasets generated or examined during this investigation are accessible from the corresponding author upon a reasonable request.
