# Supplementary material for: IL-6 knockdown anti-CD19 CAR-T cells (ssCART-19) for patients with relapsed or refractory acute lymphoblastic leukemia: phase 1 trial
Source: Blood Cancer J. 2025 Oct 27;15(1):182. doi: 10.1038/s41408-025-01397-4 (PMC12559308; doi:10.1038/s41408-025-01397-4)

**Supplementary Information**

**Supplementary Methods**

**Generation of IL‑6 shRNA‑expressing CAR constructs**

We designed short hairpin RNA (shRNA) sequences targeting the 3′ untranslated region of the human IL-6 gene using human U6 as the promotor (sequence: 5′AATTCAAAAAAGGGCACAGAACTTATGTTGTTCTCGAGAGAACAACATAAGTTCTGTGC-3′). shRNAs were synthesized by GENERAY Biotech (Shanghai, China) and inserted into a CAR construct (Unicar-Therapy Bio-medicine Technology Co., Ltd., Shanghai, China) containing a CD19-targeted single-chain variable fragment (FMC63), EF1a promoter, costimulatory 4-1BB domain, and CD3-ζ domain. The construct was co-transfected with three packaging plasmids into HEK 293T packaging cells, and the resulting lentiviruses were isolated, purified in a chromatography column, and immediately stored at −80°C.

**Manufacture of CAR-T cells**

Peripheral blood mononuclear cells from patients were separated via density gradient centrifugation, and CD3-positive T cells were enriched by positive selection using magnetic bead separation (Miltenyi Biotec, Bergisch Gladbach, Germany). These were then transduced with recombinant lentiviral vectors for 48 hours and subsequently cultured for 12–14 days in AIM-V medium (Gibco) supplemented with cytokines and autologous human serum. Testing for bacterial, fungal, and mycoplasmal contamination was performed throughout CAR-T cell preparation as well as on the day of final release of the cell product.

**Study design and participants**

**Ethical and regulatory approval**

The study protocol and informed consent form were approved by the China Center for Drug Evaluation prior to trial initiation. Approval was also obtained from the institutional review boards of all participating centers.

**Detailed definition of adequate organ function**

Adequate organ function was defined as left ventricular ejection fraction ≥50% as measured using echocardiography, serum creatinine ≤1.6 mg/dL, alanine aminotransferase or aspartate aminotransferase ≤3 times the upper limit of normal for age, total bilirubin ≤2 mg/dL, a minimum level of pulmonary reserve defined as ≤grade 1 dyspnea and pulse oxygenation >91% on room air, qualified T-cell amplification, and an estimated life expectancy of at least 3 months. Patients could not be pregnant or have plans for pregnancy within 6 months of treatment owing to concerns regarding fetal toxicity of the conditioning chemotherapy and unknown effects of CAR-T cells.

**Additional exclusion criteria**

Patients were excluded if they had isolated extra-medullary disease relapse; past or present central nervous system (CNS) diseases including epilepsy, cerebrovascular ischemia/hemorrhage, dementia, cerebellar diseases, or any CNS-related autoimmune diseases; had been treated with immunosuppressants or systemic corticosteroids within 2 weeks after signing an informed consent form; had uncontrolled bacterial, fungal, viral, mycoplasmal, or other infections; or had active acute or chronic graft-versus-host disease before providing their informed consent. Patients with over 90% CD19 expression on blasts, regardless of prior treatment with any prior anti-CD19/anti-CD3 therapy or any other anti-CD19 therapy, were eligible for study inclusion. Additional details regarding the inclusion and exclusion criteria are provided in the Supplementary protocol.

**Safety assessment and long-term follow-up**

Dose-limiting toxicity (DLT) was defined as any therapy-related grade 4 life-threatening toxicities and grade 3 or worse non-hematological toxicities, including cytokine release syndrome (CRS) that did not improve to grade 2 or lower within 7 days. Safety assessments were performed at days 28, 90, and 180 and every 6 months thereafter, up to 24 months after ssCART-19 infusion. This study was designed to follow patients for up to 15 years post CAR-T cell infusion.

**Efficacy endpoints and definitions**

Duration of response (DOR) was defined as the time from initial documentation of complete remission (CR) or CR with incomplete hematological recovery (CRi) to disease progression or death from any cause. Progression-free survival (PFS) was defined as the time from ssCART-19 infusion to disease progression or death from any cause. Overall survival (OS) was defined as the time from ssCART-19 infusion to death from any cause. CR, CRi, and disease progression were defined according to the NCCN Clinical Practice Guidelines in Oncology: Acute Lymphoblastic Leukemia, Version 2.2024.

**Pharmacokinetics and pharmacodynamics**

CAR-T cells were detected after infusion, as planned in the Supplementary protocol, to assess their amplification and persistence. CAR transgenic copies were analyzed using blood samples detected using quantitative reverse transcription polymerase chain reaction (PCR), with probe and primers targeting the sequence silencing expression of interleukin 6 (IL-6). The percentage of CAR-positive cells among CD3+ T cells was analyzed using flow cytometry. Minimal residual disease (MRD) was detected using multiparameter flow cytometry, with a positive threshold of 0.01%. A minimum of 200 000 nucleated cells with at least 20 events was analyzed to determine MRD negativity.

**Statistical analysis**

The sample size for the dose-escalation phase was determined using a standard “3+3” design to establish the recommended dose for expansion and subsequent phase 2 evaluation. For the dose-expansion cohort, the sample size was based on clinical and practical considerations to facilitate exploratory assessments of safety and preliminary efficacy. We did not have a planned number of patients for this portion of the trial. As prespecified in the protocol, safety and activity analyses were conducted for all patients who received ssCART-19 infusion.

Descriptive statistics included medians with interquartile range (IQR) for continuous variables and frequencies (counts and ratios) for categorical variables. Differences between groups were examined using an unpaired t-test, one-way analysis of variance, and Fisher’s exact test. Survival was calculated using the reverse Kaplan–Meier method. The cutoff date for all analyses was 1 April, 2025. All analyses were performed using IBM SPSS (v.22) and GraphPad Prism (v.9.0.0).

**Supplementary Results**

**Patients**

Between 9 April 2021 and 31 October 2023, we screened 31 patients. Ten patients were excluded for the following reasons: failed T-cell expansion (n=3), use of corticosteroids within 2 weeks prior to screening (n=2), CD19-negative disease (n=1), bone marrow blasts <5% at screening (n=2), elevated total bilirubin (n=1), and patient withdrawal of consent (n=1).

The median time from diagnosis to enrollment in ssCART-19 therapy was 17 months (interquartile range [IQR], 4–28). The median number of prior therapy lines was 2.0 (IQR, 1.0–2.5). Two patients had previously undergone allogeneic hematopoietic stem cell transplantation (allo-HSCT). Bridging therapy based on vindesine and dexamethasone was administered to 14 of 17 patients (82.4%) before lymphodepletion. One patient presented with grade 2 CNS leukemia at screening, evidenced by blasts in the cerebrospinal fluid and neurological symptoms including mandibular numbness, masticatory weakness, and leftward tongue deviation upon protrusion. A comprehensive summary of patients’ baseline characteristics is provided in Supplementary Table 1.

**Safety**

The most common grade 3 or higher adverse events were hematological (Supplementary Table 2). All patients achieved neutrophil recovery (≥1000/µL) and 13/17 (76.5%) achieved platelet recovery (≥50 000/µL) within 4 weeks. By day 28, 3/17 (17.6%) patients had platelet counts of 20 000/µL and recovered to the normal range by the end of month 2 post infusion. Grade 3 or higher non-hematological events related to CAR-T therapy included pyrexia (11.8%) and hypokalemia (11.8%), all of which resolved with supportive care.

Grade 3 CRS occurred in 3 of 17 patients (17.6%) whereas grade 1 and grade 2 CRS each occurred in 5 of 17 patients (29.4%) (Supplementary Table 3). The median time from ssCART-19 infusion to CRS onset was 4 days (IQR, 2–7), with a median duration 7.0 days (IQR, 4.5–8.5). In the dose-specific analysis, the median time to CRS onset was 2.0 days (IQR, 0.0–4.0), 7.0 days (IQR, 3.5–7.5), and 6.0 days for the low-, medium-, and high-dose groups, respectively. The median CRS duration was 7.0 days (IQR, 6.0–9.0), 7.0 days (IQR, 4.5–9.0), and 4.0 days for the low-, medium-, and high-dose groups, respectively.

Elevation of cytokines including interleukin 6 (IL-6), ferritin, and C-reactive protein (CRP) was observed in all patients. Peak levels occurred at a median of 7.0 days (IQR, 7.0–9.0), 10.0 days (IQR, 7.5–13.3), and 7.0 days (IQR, 4.8–9.0) post infusion in the low-, medium-, and high-dose groups, respectively. Patients in the medium-dose group exhibited higher CRP peak levels than those in the low-dose group (Supplementary Fig. 1A; P=0.0336). Peak levels of other cytokines, including IL-6, were comparable across all three dose levels (Supplementary Figs. 1B–1F). Cytokine peak levels were also comparable across all grades of CRS severity (Supplementary Fig. 2).

**Efficacy**

Two patients died following ssCART-19 infusion from septic shock due to prolonged severe neutropenia and were not evaluable for responses on day 28 or month 3. One patient with baseline severe neutropenia died on day 12 post infusion without evidence of CAR-T cell expansion. Another patient achieved CRi by day 28 but died on day 42 from a concurrent bacterial infection and COVID-19, with delayed medical intervention contributing to the outcome.

The efficacy-evaluable set (n=17) was assessed for clinical responses on day 28 and month 3 post infusion. On day 28, 14 of 17 patients (82.4%) achieved an overall response (seven CR and seven CRi), with 13 of 14 responders (92.8%) attaining MRD negativity. Among 10 patients treated with 1×10^6^ CAR-T cells/kg, 8 (80%) achieved an overall response (four CR and four CRi), all of whom were MRD-negative. By month 3, CR or CRi was sustained in 7 of the 10 patients (70%) receiving 1×10^6^ cells/kg and 4 of 6 patients (66.7%) receiving 5×10^6^ cells/kg (Supplementary Table 4).

Six patients (four in the low-dose group, one in the medium-dose group, and one in the high-dose group) experienced disease progression at a median 9.7 months (IQR, 2.9–23.4). Four patients in the low-dose group relapsed at a median 16.9 months (IQR, 8.7–25.7) post infusion. One patient in the medium-dose group relapsed at 2.2 months and died owing to disease progression. One patient had an MRD-positive relapse 1 year after ssCART-19 infusion, initially responded to inotuzumab ozogamicin, but subsequently relapsed 26.8 months after initial treatment.

Notably, one patient presented with grade 2 CNS leukemia and symptomatic infiltration at screening, with 5.1% blasts in cerebrospinal fluid (CSF) and 66% blasts in bone marrow. The CSF blasts cleared following two cycles of intrathecal chemotherapy with methotrexate, cytarabine, and dexamethasone. The patient achieved CR by day 28 and underwent allo-HSCT 7.6 months post infusion.

**Pharmacokinetics**

We detected expansion of CAR-T cells in all patients (Supplementary Fig. 4 and 5). The median maximum CAR-T cell expansion (C_max_) was 31 400 CAR gene copies/μg DNA (IQR, 5090**–**140 500). The median C_max_ and the area under the curve during the first 28 days following CAR-T cell infusion (AUC_0_**_–_**_28d_) were 45 400 and 375 094 in the low-dose group, 52 035 and 449 814 in the medium-dose group, 9790 and 29 646 in the high-dose group (Supplementary Table 5). The median time to reach C_max_ was 11.0 days (IQR, 11.0**–**14.0).

| **Patient ID** | **Age, years** | **Sex** | **Refractory** | **Relapse** | **ECOG score** | **Cytogenetics** | **Mutations** | **Risk**  **classification** | **Time since diagnosis, months** | **CNS disease** | **Blasts in BM at screening** | **Blasts in BM before infusion** | **CD19**  **expression in blasts** | **Previous HSCT** | **Bridging therapy** |
| --- | --- | --- | --- | --- | --- | --- | --- | --- | --- | --- | --- | --- | --- | --- | --- |
| **S002** | 18 | M | N | Y | 1 | (-) | (-) | Standard | 4 | N | 17.00% | 2.00% | 100.00% | N | Y |
| **S006** | 20 | F | N | Y | 0 | (-) | *SH2B3*; *IL7R* | Poor | 29 | N | 15.60% | 10.00% | 94.40% | Y | Y |
| **S007** | 19 | M | N | Y | 0 | (-) | (-) | Standard | 26 | N | 77.00% | 1.00% | 100.00% | N | Y |
| **S008** | 18 | F | Y | N | 0 | (-) | *EPOR* | Poor | 2 | N | 14.00% | 32.00% | 100.00% | N | N |
| **S011** | 35 | F | N | Y | 1 | (-) | (-) | Standard | 29 | N | 63.50% | 2.00% | 100.00% | N | Y |
| **S012** | 47 | F | N | Y | 1 | Complex karyotype | *EZH2*; *FLT3*; *TP53* | Poor | 2 | Y | 66.00% | 59.50% | 100.00% | N | Y |
| **S015** | 42 | M | N | Y | 0 | t(14.18) (q32.q21), -16, +mar, inc [2] | *PAX5* | Poor | 3 | N | 87.00% | 40.00% | 94.30% | N | Y |
| **S016** | 39 | M | Y | N | 0 | *NUP214-ABL1* fusion | *SETD2* | Standard | 2 | N | 7.00% | 2.50% | 21.79% | N | N |
| **S017** | 53 | F | Y | N | 1 | (-) | (-) | Standard | 5 | N | 8.00% | 8.00% | 100.00% | N | N |
| **S018** | 46 | M | N | Y | 1 | 14q+ [1] | *PTPN*; *RUNX1*; *KDM6A*; *PAX5* | Poor | 6 | N | 23.50% | 23.00% | 100.00% | N | Y |
| **S020** | 52 | F | N | Y | 1 | (-) | (-) | Standard | 18 | N | 76.50% | 49.00% | 100.00% | N | Y |
| **S022** | 61 | F | N | Y | 1 | *BCR::ABL1* fusion | (-) | Standard | 25 | N | 31.00% | 12.00% | 100.00% | N | Y |
| **S027** | 19 | M | N | Y | 1 | *TEL::AML1* fusion | (-) | Standard | 204 | N | 68.50% | 18.00% | 100.00% | N | Y |
| **S028** | 39 | M | N | Y | 1 | *BCR::ABL1* fusion | (-) | Standard | 60 | N | 88.00% | 57.50% | 100.00% | N | Y |
| **S029** | 49 | M | N | Y | 1 | (-) | *KMT2D*; *PWWP3A*; *RELN* | Standard | 14 | N | 69.50% | 79.00% | 100.00% | N | Y |
| **S030** | 58 | F | N | Y | 1 | (-) | (-) | Standard | 27 | N | 7.00% | 61.50% | 99.17% | N | Y |
| **S031** | 34 | M | N | Y | 1 | *BCR::ABL1* fusion | (-) | Standard | 17 | N | 17.00% | 7.50% | 93.70% | Y | Y |

**Supplementary Table 1: Detailed baseline characteristics of patients**

**Supplementary Table 2: Adverse events**

| **Adverse events, n (%)** | **Grade 1** | **Grade 2** | **Grade 3** | **Grade 4** |
| --- | --- | --- | --- | --- |
| **Hematological** |  |  |  |  |
| Lymphopenia | 0 | 0 | 2 (11.8) | 14 (82.4) |
| Neutropenia | 0 | 1 (5.9) | 2 (11.8) | 13 (76.5) |
| Leukopenia | 0 | 1 (5.9) | 2 (11.8) | 13 (76.5) |
| Anemia | 0 | 4 (23.5) | 12 (70.6) | 0 |
| Thrombocytopenia | 4 (23.5) | 1 (5.9) | 2 (11.8) | 8 (47.1) |
| Febrile neutropenia | 0 | 0 | 1 (5.9) | 0 |
| **Gastrointestinal disorders** |  |  |  |  |
| Diarrhea | 1 (5.9) | 0 | 0 | 0 |
| Nausea | 4 (23.5) | 0 | 0 | 0 |
| Vomiting | 1 (5.9) | 1 (5.9) | 0 | 0 |
| Abdominal pain | 1 (5.9) | 0 | 0 | 0 |
| **General disorders** |  |  |  |  |
| Fatigue | 1 (5.9) | 0 | 0 | 0 |
| Asthenia | 1 (5.9) | 0 | 0 | 0 |
| Pyrexia | 6 (35.3) | 5 (29.4) | 2 (11.8) | 0 |
| Hypotension | 3 (17.6) | 2 (11.8) | 1 (5.9) | 0 |
| **Metabolism and nutritional disorders** |  |  |  |  |
| Decreased appetite | 1 (5.9) | 0 | 0 | 0 |
| Hypokalemia | 0 | 11 (64.7) | 2 (11.8) | 0 |
| Hypocalcemia | 4 (23.5) | 7 (41.2) | 1 (5.9) | 0 |
| Hypophosphatemia | 9 (52.9) | 0 | 0 | 0 |
| Hypoglobulinemia | 13 (76.5) | 0 | 0 | 0 |
| Hypogammaglobulinemia | 8 (47.1) | 0 | 0 | 0 |
| **Nervous system disorders** |  |  |  |  |
| Headache | 2 (11.8) | 0 | 0 | 0 |
| Dizziness | 2 (11.8) | 0 | 0 | 0 |
| **Respiratory diseases** |  |  |  |  |
| Cough | 2 (11.8) | 0 | 0 | 0 |
| Productive cough | 1 (5.9) | 0 | 0 | 0 |
| **Investigation results** |  |  |  |  |
| Increased aspartate aminotransferase | 9 (52.9) | 1 (5.9) | 1 (5.9) | 0 |
| Increased alanine aminotransferase | 9 (52.9) | 2 (11.8) | 0 | 0 |
| Hypofibrinogenemia | 4 (23.5) | 0 | 1 (5.9%) | 0 |
| Prolonged prothrombin time | 3 (17.6) | 0 | 0 | 0 |
| **CRS** | 5 (29.4) | 5 (29.4) | 3 (17.6) | 0 |
| **ICANS** | 0 | 0 | 0 | 0 |

**Supplementary Table 3: Summary of CRS and ICANS**

|  | **1×10^6^/kg (N=10)** | **5×10^6^/kg (N=6)** | **1×10^7^/kg (N=1)** | **All patients (N=17)** |
| --- | --- | --- | --- | --- |
| **CRS, n (%)** | | | | |
| Any grade | 7 (70.0) | 5 (83.3) | 1 (100.0) | 13 (76.5) |
| Grade 1 | 3 (30.0) | 1 (16.7) | 1 (100.0) | 5 (29.4) |
| Grade 2 | 3 (30.0) | 2 (33.3) | 0 | 5 (29.4) |
| Grade 3 | 1 (10.0) | 2 (33.3) | 0 | 3 (17.6) |
| **Median time to onset** | | | | |
| n (%) | 7 (70.0) | 5 (83.3) | 1 (100.0) | 13 (76.5) |
| Mean (SD) | 2.7 (3.1) | 5.8 (2.4) | 6.0 | 4.1 (3.1) |
| Median | 2.0 | 7.0 | - | 4.0 |
| Days (IQR) | 0.0**–**4.0 | 3.5**–**7.5 | - | 2.0**–**7.0 |
| **Median duration of CRS** | | | | |
| n (%) | 7 (70.0) | 5 (83.3) | 1 (100.0) | 13 (76.5) |
| Mean (SD) | 7.4 (2.2) | 6.8 (2.4) | 4.0 | 6.9 (2.3) |
| Median | 7.0 | 7.0 | - | 7.0 |
| Days (IQR) | 6.0**–**9.0 | 4.5**–**9.0 | - | 4.5**–**8.5 |
| **Therapy for CRS, n (%)** | | | | |
| Tocilizumab | 6 (60.0) | 3 (50.0) | 0 | 9 (52.9) |
| Corticosteroids | 5 (50.0) | 3 (50.0) | 0 | 8 (47.1) |
| **ICANS, n (%)** | | | | |
| Any grade | 0 | 0 | 0 | 0 |

CRS=cytokine release syndrome. ICANS=immune effector cell-associated neurotoxicity syndrome. SD=standard deviation. IQR=interquartile range.

**Supplementary Table 4: Treatment response stratification by ssCART-19 dose cohort**

| **Response rates at day 28, n (%)** | **1×10^6^/kg (N=10)** | **5×10^6^/kg**  **(N=6)** | **1×10^7^/kg (N=1)** | **All patients (N=17)** |
| --- | --- | --- | --- | --- |
| **ORR** | 8 (80.0) | 5 (83.3) | 1 (100.0) | 14 (82.4) |
| **CR** | 4 (40.0) | 3 (50.0) | 0 | 7 (41.2) |
| **CRi** | 4 (40.0) | 2 (33.3) | 1 (100.0) | 7 (41.2) |
| **MRD+ CR** | 0 | 1 (20.0) | 0 | 1 (7.1) |
| **MRD- CR** | 8 (100.0) | 4 (80.0) | 1 (100.0) | 13 (92.9) |
| **NR** | 1 (10.0) | 1 (16.7) | 0 | 2 (11.8) |
| **Response rates at month 3, n (%)** | **1×10^6^/kg (N=10)** | **5×10^6^/kg (N=6)** | **1×10^7^/kg (N=1)** | **All patients (N=17)** |
| **ORR** | 7 (70.0) | 4 (66.7) | 0 | 11 (64.7) |
| **CR** | 5 (50.0) | 3 (50.0) | 0 | 8 (47.1) |
| **CRi** | 2 (20.0) | 1 (16.7) | 0 | 3 (17.6) |
| **MRD+ CR** | 0 | 0 | 0 | 0 |
| **MRD- CR** | 7 (100.0) | 4 (100.0) | 0 | 11 (100.0) |
| **NR** | 1 (10.0) | 2 (33.3) | 1 (100.0) | 4 (23.5) |

**Note:** One patient in the low-dose group died on day 12 from septic shock due to prolonged and severe neutropenia and was therefore excluded from the efficacy analysis at day 28 post infusion. Another patient in the low-dose group died on day 41 from septic shock and consequently lacked efficacy assessment results at 3 months post infusion.

ORR=objective response rate. CR=complete response. CRi=CR with incomplete hematological recovery. MRD=minimal residual disease. NR=no response.

**Supplementary Table 5: Summary of ssCART-19 kinetics**

|  | 1×10**^6^**/kg  (N=10) | 5×10**^6^**/kg  (N=6) | 1×10**^7^**/kg  (N=1) | **All patients**  **(n=17)** |
| --- | --- | --- | --- | --- |
| **C_max_ (CAR gene copies per μg DNA)** | | | | |
| Median | 45 400 | 52 035 | 9790 | 31 400 |
| IQR | 2730**–**1 387 500 | 5539**–**246 000 | - | 5090**–**140 500 |
| **T_max_ (days)** | | | | |
| Median | 11.0 | 12.5 | 9.0 | 11.0 |
| IQR | 11.0**–**14.0 | 10.5**–**14.0 | - | 11.0**–**14.0 |
| AUC**_0-28d_** (CAR gene copies **per μg DNA**) | | | | |
| Median | 375 094 | 449 814 | 29646 | 324 325 |
| IQR | 30 269**–**  1 059 737 | 17 528**–**  1 785 233 | - | 26 361**–**  987 055 |

CAR=chimeric antigen receptor. C_max_=maximum CAR-T cell expansion. T_max_=time to peak CAR-T cell expansion. AUC_0-28_=area under the receiver operating characteristic curve during the first 28 days following CAR-T cell infusion. IQR=interquartile range.

**Supplementary Figure 1: Association between serum inflammatory marker levels and dose**


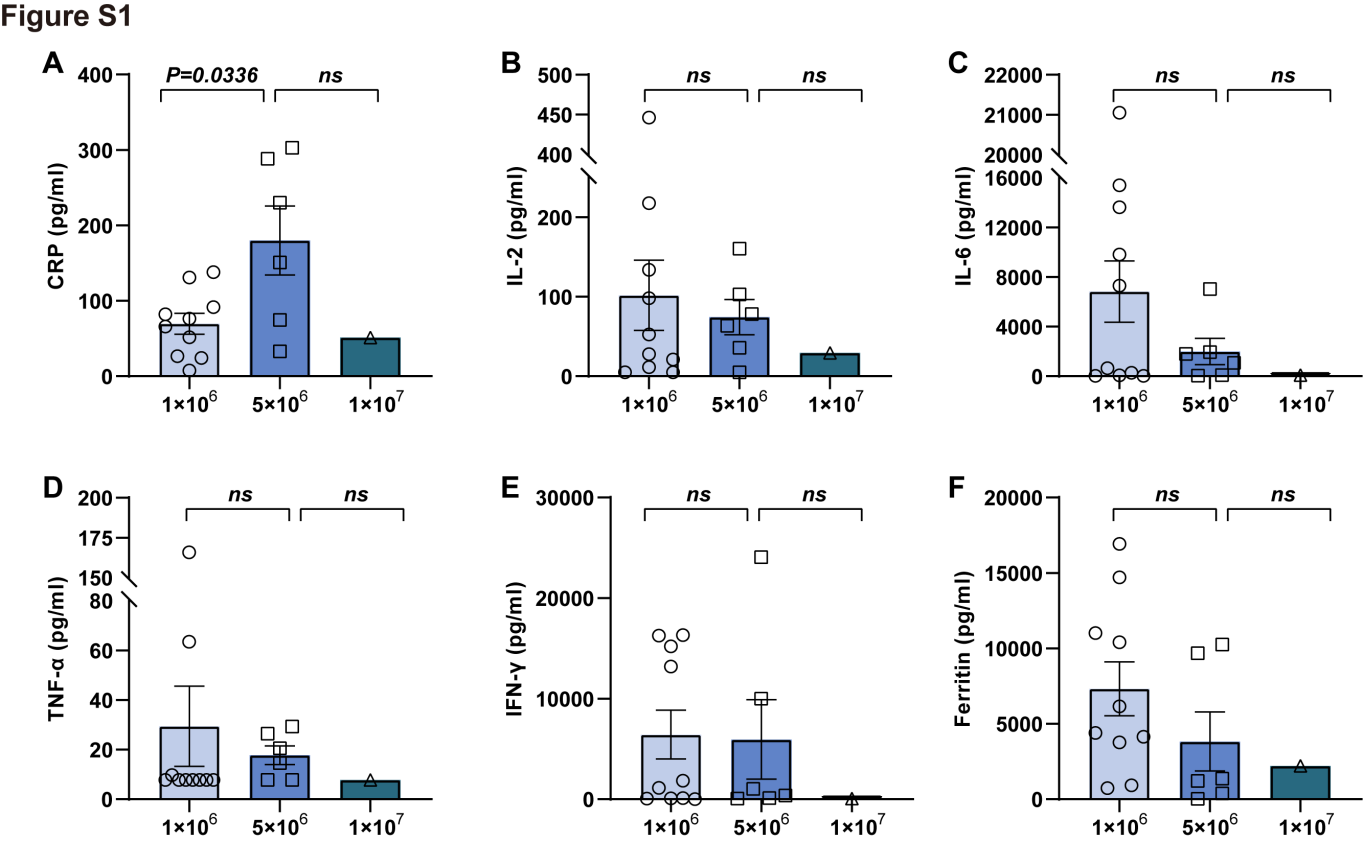


**Supplementary Figure 2: Association between serum inflammatory marker levels and CRS grades**


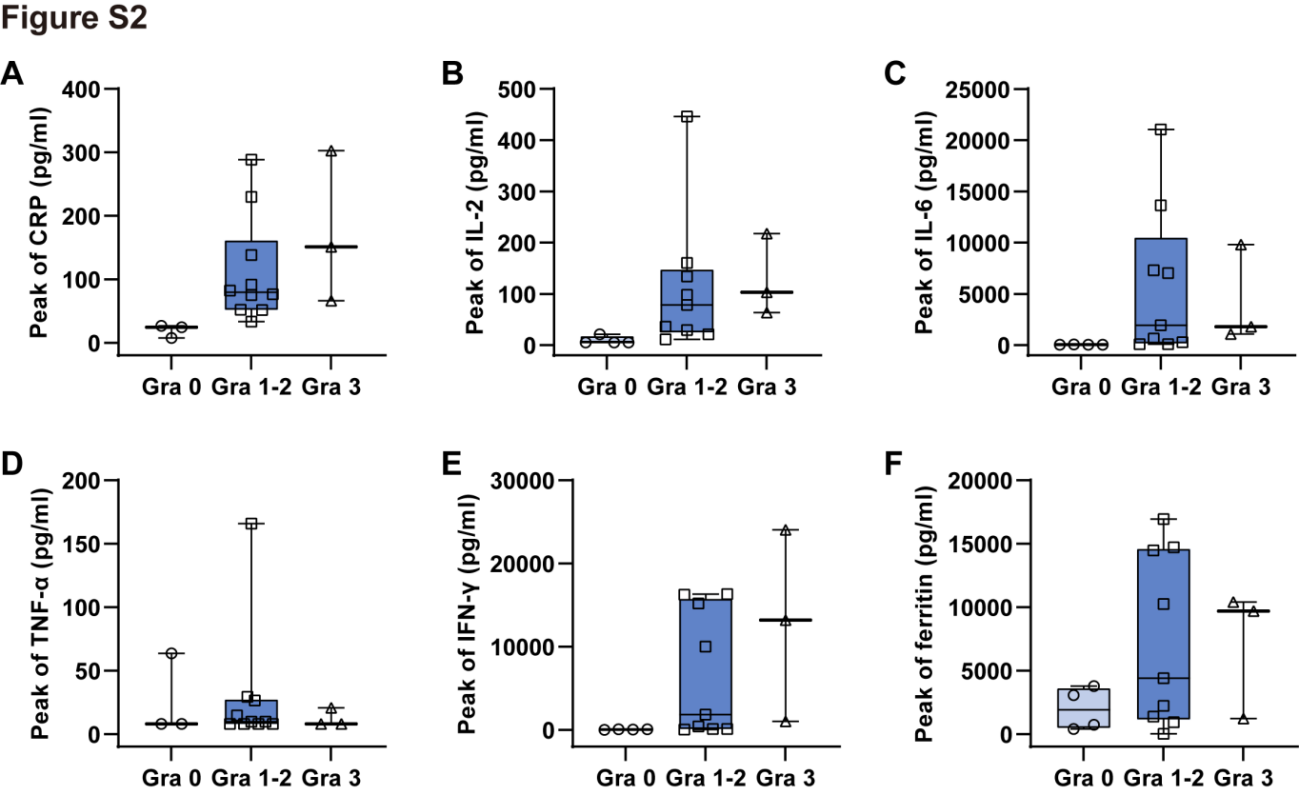


**Supplementary Figure 3: Patient survival analysis**

(A) The median duration of response (DOR) for the 14 patients who achieved CR or CRi by day 28 after ssCART-19 infusion was 25.8 months (range, 4.8**–**46.9). (B) The median progression-free survival (PFS) was 22.2 months (range, 0.0**–**47.0). (C) The median overall survival (OS) was not reached by the cutoff date. NE=not estimable. NR=not reached.


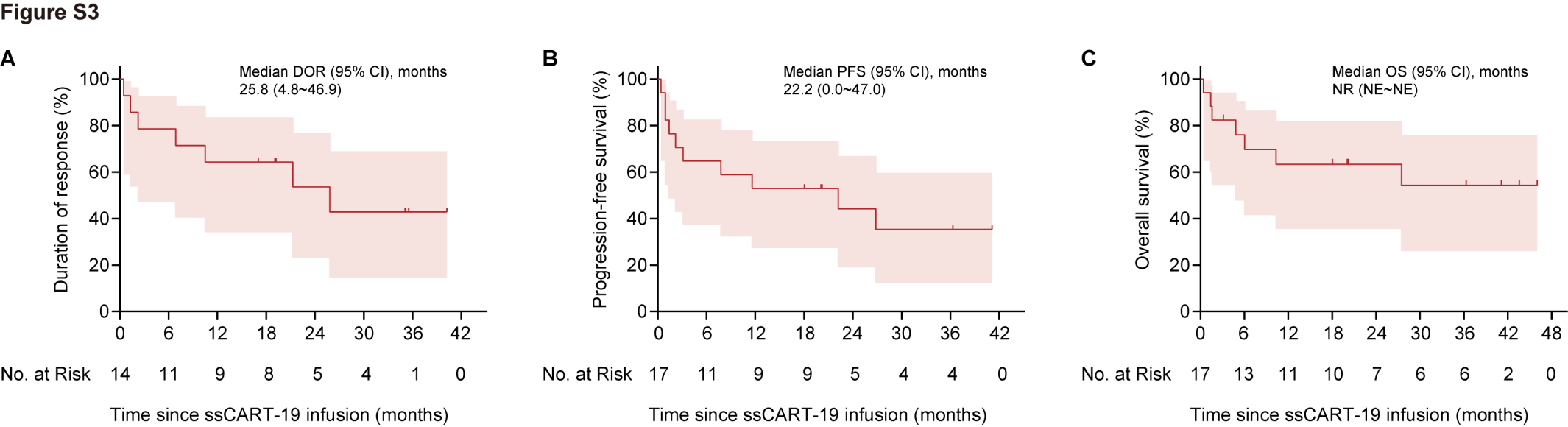


**Supplementary Figure 4: Kinetics of CAR-T cells in peripheral blood**

(A) CAR gene copies per μg DNA measured using quantitative PCR. (B) Percentage of CAR-positive cell among CD3+ T cells measured using flow cytometry. PB=peripheral blood.


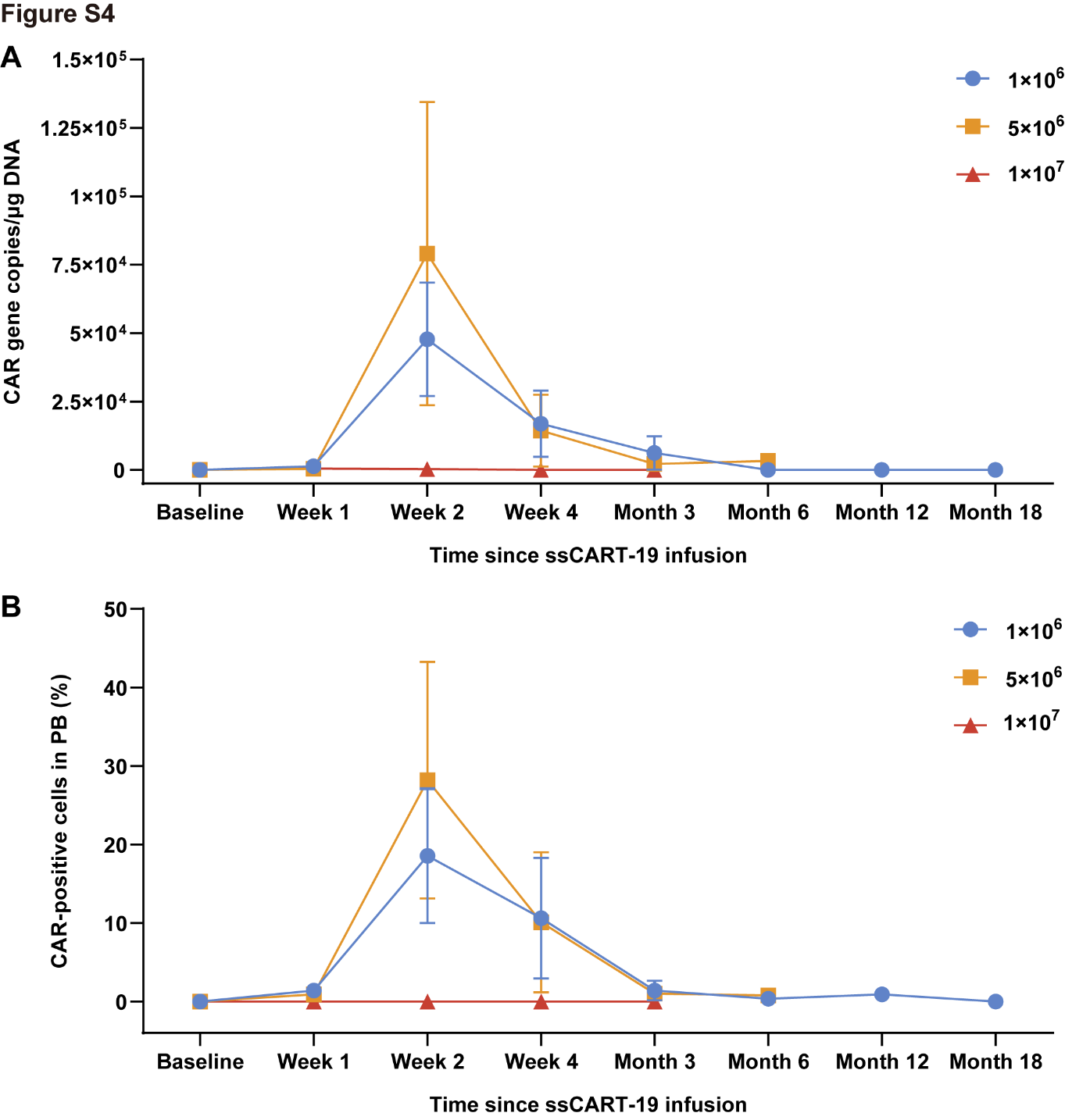


**Supplementary Figure 5: Kinetics of CAR-T cells in patients with different responses**

(A, B) Levels of CAR-T cell expansion in peripheral blood measured using quantitative PCR and flow cytometry in patients with r/r B-ALL. (C–E) Association between CAR-T cell expansion and responses at month 3 after ssCART-19 infusion.


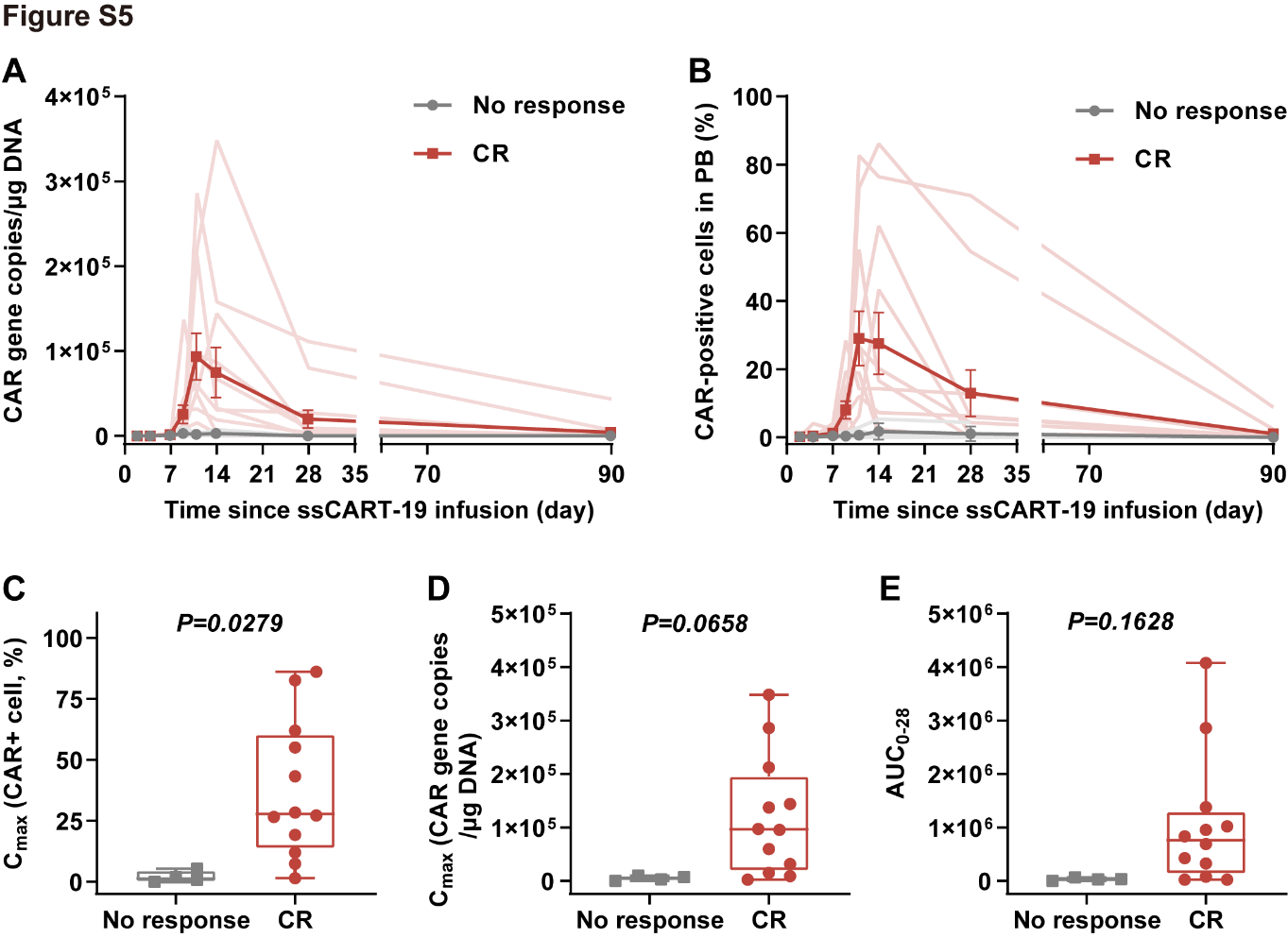

Supplement: Supplementary file 1 — Supplementary Information [file 41408_2025_1397_MOESM1_ESM.docx]
